# Are infectious diseases risk factors for sarcoidosis or a result of reverse causation? Findings from a population-based nested case–control study

**DOI:** 10.1007/s10654-020-00611-w

**Published:** 2020-02-11

**Authors:** Marios Rossides, Susanna Kullberg, Johan Askling, Anders Eklund, Johan Grunewald, Daniela Di Giuseppe, Elizabeth V. Arkema

**Affiliations:** 1 Clinical Epidemiology Division, Department of Medicine Solna, Karolinska Institutet, Karolinska University Hospital, Eugeniahemmet T2, 171 76 Stockholm, Sweden; 2 Respiratory Medicine Division, Department of Medicine Solna, Karolinska Institutet, Stockholm, Sweden; 3 Center for Molecular Medicine, Karolinska Institutet, Stockholm, Sweden; 4 Respiratory Medicine, Theme Inflammation and Infection, Karolinska University Hospital, Stockholm, Sweden; 5 Rheumatology, Theme Inflammation and Infection, Karolinska University Hospital, Stockholm, Sweden

**Keywords:** Sarcoidosis, Infection, Case–control study, Etiology, Reverse causation

## Abstract

Findings from molecular studies suggesting that several infectious agents cause sarcoidosis are intriguing yet conflicting and likely biased due to their cross-sectional design. As done in other inflammatory diseases to overcome this issue, prospectively-collected register data could be used, but reverse causation is a threat when the onset of disease is difficult to establish. We investigated the association between infectious diseases and sarcoidosis to understand if they are etiologically related. We conducted a nested case–control study (2009–2013) using incident sarcoidosis cases from the Swedish National Patient Register (n = 4075) and matched general population controls (n = 40,688). Infectious disease was defined using inpatient/outpatient visits and/or antimicrobial dispensations starting 3 years before diagnosis/matching. Adjusted odds ratios (aOR) of sarcoidosis were estimated using conditional logistic regression and tested for robustness assuming the presence of reverse causation bias. The aOR of sarcoidosis associated with history of infectious disease was 1.19 (95% confidence interval [CI] 1.09, 1.29; 21% vs. 16% exposed cases and controls, respectively). Upper respiratory and ocular infections conferred the highest OR. Findings were similar when we altered the infection definition or varied the infection-sarcoidosis latency period (1–7 years). In bias analyses assuming one in 10 infections occurred because of preclinical sarcoidosis, the observed association was completely attenuated (aOR 1.02; 95% CI 0.90, 1.15). Our findings, likely induced by reverse causation due to preclinical sarcoidosis, do not support the hypothesis that common symptomatic infectious diseases are etiologically linked to sarcoidosis. Caution for reverse causation bias is required when the real disease onset is unknown.

**Electronic supplementary material:**

The online version of this article (10.1007/s10654-020-00611-w) contains supplementary material, which is available to authorized users.

## Introduction

The etiology of sarcoidosis remains to this day a mystery [1]. The contemporary notion entails that an unidentified environmental factor triggers immune disturbance in a genetically susceptible individual [1]. This disturbance leads to systemic granulomatous inflammation, which predominantly affects the pulmonary and lymphatic systems [2]. Infectious agents are perceived to be likely candidates for explaining the environmental fraction of disease etiology [2], which was estimated to be accountable for about 61% of the susceptibility to disease [3].

In fact, infectious agents gained the most research attention throughout the years largely due to the clinical and histological similarities between sarcoidosis and tuberculosis. Except mycobacteria [4, 5], the role of other bacteria such as propionibacteria [6–9], as well as viruses [10], fungi [11, 12] and the lung microbiome overall [13, 14] has been investigated in numerous molecular studies. The causal role of these infectious agents is still obscure as results are conflicting. Most importantly, samples for molecular analysis were obtained after sarcoidosis diagnosis rendering any conclusions prone to reverse causation bias, that is, sarcoidosis leading to infectious disease rather than the opposite.

Findings from epidemiological assessments are equally conflicting. Exposure to moldy environments rich in aerosolized infectious agents [15], and the geographical [16, 17] and seasonal variation of disease occurrence [18] are suggestive of the etiological implication of infectious agents. However, antimicrobial treatment is not generally used to achieve disease remission [2, 19]. Except for a small Taiwanese study suggesting an eightfold increased rate of sarcoidosis associated with tuberculosis [20], no epidemiologic investigations have examined whether infectious disease is associated with sarcoidosis development. In several other inflammatory diseases for which the true onset of disease is unknown [21–23], prospectively-collected register data have been used to elucidate on the role of infection in their etiology, but how reverse causation might explain any association found has not been extensively investigated.

Quantifying the potential contribution of infectious disease to the etiology of sarcoidosis has profound implications for clinical practice and research, facilitating diagnosis and treatment and guiding future research efforts. To determine whether infectious disease could be etiologically linked to sarcoidosis development, we performed a nested case–control study using information derived from Swedish population-based registers. Our objective was to estimate relative risks of sarcoidosis associated with a history of infectious disease, overall, by sarcoidosis phenotype, type, and temporal proximity of infectious disease to sarcoidosis diagnosis and test the robustness of our findings in the presence of reverse causation bias.

## Methods

### Sarcoidosis cases and general population controls

We conducted a case–control study nested in the Swedish population (2009–2013). We used the National Patient Register (NPR) to identify individuals with sarcoidosis. The NPR holds high quality data on hospitalizations (nationwide since 1987) and outpatient visits to specialists in public and private practices since 2001. Visits were coded using the International Classification of Diseases (ICD) system [24]. Individuals with at least two inpatient or outpatient visits listing an ICD code for sarcoidosis in the NPR between Jan 1, 2009 and Dec 31, 2013 were classified as sarcoidosis cases. Codes are listed in Table S1 in the Supplement. Requiring a washout period of at least 8 years since the inception of NPR’s outpatient component (in 2001) allowed us to capture newly diagnosed individuals. To minimize sarcoidosis misclassification, we excluded individuals younger than 18 years and those with a hematopoietic or lung malignancy registered in the Cancer Register within 6 months before or after the first visit for sarcoidosis (index date).

Individuals with progressive sarcoidosis or debilitating symptoms receive pharmacologic treatment, commonly, systemic corticosteroids, methotrexate, or azathioprine in Sweden [25, 26]. Because there is no information on sarcoidosis severity in the register data, we used a previously developed proxy [27] which defines severe sarcoidosis phenotype as cases who were dispensed any of the three treatments in the Prescribed Drug Register within 3 months before or after the index date (Table S1). The Prescribed Drug Register captures all prescription dispensations in pharmacies across the country since July 2005. In addition, we could obtain information on disease phenotype, i.e. Löfgren’s syndrome or non-Löfgren’s disease for 324 individuals who were registered in our clinical cohort at Karolinska University Hospital in Stockholm. Löfgren’s syndrome (bihilar lymphadenopathy, erythema nodosum, and/or periarticular ankle swelling) presents with acute symptomatology, but is likely to resolve within 2 years [28].

Up to 10 controls per case were sampled from the general population at index date from the Total Population Register and matched on birth year, sex, and residential location. Only controls who lived in Sweden at the date their matched case had their second visit for sarcoidosis and had no hematopoietic or lung malignancy within 6 months before or after the index date were included in the analyses.

Ethical approval for this study was granted by the Regional Ethics Review Board in Stockholm (Decision No. 2014/230-31).

### History of infectious disease and latency period

History of infectious disease was defined using information on visits in the NPR listing an ICD code for infectious disease and/or antimicrobial dispensations in the Prescribed Drug Register. Thus, only symptomatic infections listed in Table S1 that led to an interaction with healthcare were captured by this exposure definition. The primary definition was at least one inpatient or outpatient visit listing an ICD code for infectious disease as primary or contributory discharge diagnosis. To approximate infectious disease severity and reduce misclassification, we restricted to hospitalizations, or to visits where infection was the primary discharge diagnosis, or required two or more visits for infection. To capture infections diagnosed and treated solely in primary care, we expanded the primary definition to include at least one dispensation of an antimicrobial medication.

**Fig. 1:**
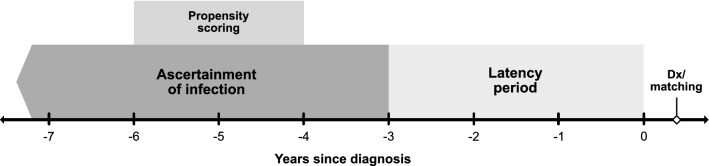
Graphical presentation of the study design. Individuals were required to have two visits for sarcoidosis in the National Patient Register; “0” indicates the first visit and “Dx” the second visit for sarcoidosis in the register

Sarcoidosis at a preclinical stage may last for more than 2 years before diagnosis in some cases [26, 29–31], hence increasing the risk for reverse causation bias. To mitigate the possibility of capturing infectious diseases occurring while cases were at the preclinical stage, visits or dispensations occurring within 3 years before index date in both cases and controls were not considered in main analyses (Fig. 1).

### Other variables

We obtained demographic information from the Total Population Register including the birth date (to calculate age), sex, birth country (Nordic, non-Nordic, missing), civil status (registered as living with partner, living alone, missing), and county of residence at diagnosis/matching, which we classified into six healthcare regions (Stockholm, Uppsala-Örebro, West, South, Southeast, and North). From the Longitudinal Integration Database for Health Insurance and Labor Market Studies, we retrieved data on education (≤ 9, 10‒12, ≥ 13 years, missing) and annual gross salary adjusted to 2014 inflation rate [32] of the year before the start of exposure ascertainment, that is, at least 3 years before sarcoidosis diagnosis/matching for main analyses (< 100, 100–< 300, 300–< 600, ≥ 600 thousand Swedish krona, missing).

We hypothesized that a higher genetic susceptibility to or the presence of a comorbid autoimmune disease in sarcoidosis might confound the association between history of infection and sarcoidosis development [33]. To adjust for these, we searched for history of autoimmune disease in both cases and controls and their first degree relatives identified in the Multi-Generation Register. We defined history of autoimmune disease as at least one visit listing a code for such disease in the NPR (Table S1).

### Statistical analysis

Using conditional logistic regression models, we estimated odds ratios (OR) for sarcoidosis associated with a history of infectious disease. We reported ORs adjusted for matching factors and ORs further adjusted for deciles of a high-dimensional propensity score estimated separately for each analysis. As detailed in the Supplement, we used a semi-automated algorithm [34] and data on healthcare visits and prescription dispensations to construct and select potential confounding covariates defined over a two-year period starting 1 year before exposure ascertainment (Fig. 1). Restricting to data within a two-year period allowed us to capture morbidity in all individuals irrespective of the year of study entry and balance computational complexity. In addition to covariates identified by the algorithm, predefined demographic and clinical confounding variables were also used in the estimation of the propensity scores. These variables were evaluated at exposure ascertainment (i.e. 3 years before sarcoidosis diagnosis or matching unless otherwise stated) and were not restricted to the time span used for algorithm-derived covariates.

We performed several other analyses. First, we calculated the population attributable fraction (as outlined in the Supplement) to assess the contribution of infectious disease to sarcoidosis occurrence. Second, we examined the odds of sarcoidosis conferred by specific types of infection (respiratory, skin, ocular, gastrointestinal, and genitourinary; Table S1). In addition, we used acne as a proxy of infection by propionibacteria to corroborate reports on their role in sarcoidosis, and urinary tract infections as negative control. Third, we varied the latency period from 0 to 7 years to investigate whether there was effect measure modification by the time since infectious disease diagnosis. Last, to examine if ORs varied by sarcoidosis severity around diagnosis or phenotype, we estimated the association separately for treated and not treated sarcoidosis, and for Löfgren’s and non-Löfgren’s disease, the latter using data from our clinical cohort.

Despite the fact that exposure and outcome were collected at least 3 years apart, a critical concern was differential exposure misclassification (reverse causation bias). That is, compared to the mostly healthy controls, individuals who were diagnosed with sarcoidosis were more likely to receive the diagnosis of infection due to undiagnosed preclinical sarcoidosis. Another concern was potential confounding of the association by smoking, data for which we did not have. To check the robustness of the conventional analyses against these two systematic errors, we performed a series of probabilistic bias analysis described in detail in the Supplement. In brief, we estimated bias-corrected ORs from three simulation analyses for differential exposure misclassification and from one simulation for unmeasured confounding. For reverse causation bias, we opted for three bias scenarios to account for the uncertainty around the bias parameters because data on the proportion of cases that had preclinical sarcoidosis were not available [26].

Data were managed and analyzed using SAS software version 9.4 (SAS Institute Inc., Cary, NC, USA) and R version 3.5.3 (R Foundation for Statistical Computing, Vienna, Austria).

## Results

We included 4075 newly diagnosed sarcoidosis cases and 40 688 matched general population controls. Cases and controls were on average 51 years old (SD 15.2), 45% were female, and comparable with respect to birth country, civil status, education, and salary (Table 1). Compared to controls and their first degree relatives, cases and their relatives were more likely to have a history of autoimmune disease (8 vs. 12% in participants and 42 vs. 47% in relatives, respectively).

**Table 1:** Baseline demographic and clinical characteristics of sarcoidosis cases and general population controls

	Sarcoidosis cases	General population controls
Individuals, n	4075	40 688
Age at diagnosis/matching, mean (SD)	51 (15.2)	51 (15.2)
Female, %	44.5	44.5
Region of residence, %		
Stockholm	20.4	20.5
Uppsala-Örebro	21.7	21.6
West	17.3	17.3
South	17.3	17.3
Southeast	11.8	11.7
North	11.5	11.5
Missing	< 0.1	0.1
Country of birth^a^, %		
Nordic	89.0	86.7
Non-Nordic	10.7	12.9
Missing	0.3	0.4
Years of education^b^, %		
≤ 9	20.5	20.4
10‒12	48.9	45.8
≥ 13	27.9	31.1
Missing	2.7	2.8
Annual gross salary in 1000 SEK^c^, %		
< 100	38.8	38.3
100‒ < 300	28.6	28.9
300‒ < 600	28.1	27.8
≥ 600	2.7	3.4
Missing	1.7	1.6
Registered as living with partner^b^, %		
Yes	48.6	48.4
No	51.3	51.6
Missing	< 0.1	0.1
History of autoimmune disease^b^, %	12.4	8.2
At least one first degree relative with autoimmune disease or sarcoidosis, %	47.3	42.5
Sarcoidosis in need of treatment at diagnosis, %	42.0	–

Percentages may not sum to 100 owing to rounding

*SD* standard deviation, *SEK* Swedish krona

^a^Nordic countries include Sweden, Denmark, Norway, Finland, and Iceland

^b^Evaluated at the year before exposure ascertainment (4 years before sarcoidosis diagnosis or matching)

^c^Refers to the salary earned during the year before exposure ascertainment (4 years before sarcoidosis diagnosis or matching) adjusted for 2014 inflation level. 1.00 SEK ≈ 0.10 USD, 0.09 EUR, or 0.08 GBP

After allowing for a lag time of 3 years between exposure ascertainment and sarcoidosis diagnosis or matching, we found that 21% of cases and 16% of controls had at least one visit for infection (Table 2). After adjustment for confounders, which resulted in substantial attenuation of the association, the OR of sarcoidosis associated having a visit for infectious disease was 1.19 (95% CI 1.09, 1.29) resulting in a population attributable fraction of 3.3% (95% CI 1.7%, 4.8%). The magnitude of the association did not materially change when we required at least two visits for infection, when we restricted to hospitalizations, primary diagnoses, or when we counted dispensations of antimicrobials in addition to visits for infection (Table 2).

**Table 2:** Odds ratios of sarcoidosis associated with a history of infectious disease defined by ICD codes from visits in the National Patient Register and/or dispensations of antimicrobials in the Prescribed Drug Register, by definition and type of infectious disease

	History of infectious disease, n (%)		Odds ratio of sarcoidosis (95% CI)	
	Sarcoidosis cases (n = 4075)	General population controls (n = 40 688)	Adjusted for matching factors	Adjusted for high-dimensional propensity score^a^
*Definition of infectious disease*				
≥ 1 visit for infectious disease				
Overall	846 (20.8)	6461 (15.9)	1.40 (1.29, 1.52)	1.19 (1.09, 1.29)
Hospitalizations only	346 (8.5)	2503 (6.2)	1.42 (1.26, 1.60)	1.24 (1.10, 1.39)
Primary diagnoses only	762 (18.7)	5709 (14.0)	1.42 (1.31, 1.55)	1.21 (1.11, 1.32)
Hospitalizations and primary diagnoses	280 (6.9)	1956 (4.8)	1.47 (1.29, 1.67)	1.26 (1.11, 1.43)
≥ 2 visits for infectious disease	372 (9.1)	2520 (6.2)	1.53 (1.37, 1.72)	1.28 (1.14, 1.44)
≥ 1 visit for infectious disease or ≥ 1 dispensation of antimicrobials	2260 (55.5)	19 589 (48.1)	1.38 (1.29, 1.48)	1.21 (1.13, 1.30)
*Type of infectious disease*^b^				
Respiratory^c^				
Overall	280 (6.9)	1878 (4.6)	1.54 (1.35, 1.75)	1.25 (1.10, 1.42)
Upper only	171 (4.2)	1153 (2.8)	1.51 (1.28, 1.79)	1.30 (1.10, 1.53)
Lower only	116 (2.8)	850 (2.1)	1.38 (1.13, 1.68)	1.12 (0.93, 1.36)
Skin^d^				
Overall	319 (7.8)	2580 (6.3)	1.26 (1.11, 1.42)	1.15 (1.02, 1.29)
Acne only	63 (1.5)	625 (1.5)	1.01 (0.77, 1.31)	0.96 (0.74, 1.25)
Ocular	12 (0.3)	72 (0.2)	1.67 (0.90, 3.08)	1.93 (1.12, 3.33)
Gastrointestinal	120 (2.9)	940 (2.3)	1.29 (1.06, 1.56)	1.15 (0.95, 1.39)
Genitourinary				
Overall	267 (6.6)	2202 (5.4)	1.24 (1.08, 1.41)	1.06 (0.92, 1.21)
Urinary tract only	95 (2.3)	647 (1.6)	1.49 (1.19, 1.85)	1.24 (1.00, 1.54)

A latency period between infectious disease ascertainment and sarcoidosis diagnosis/matching of at least 3 years was required

*CI* confidence interval

^a^Adjusted for deciles of a high-dimensional propensity score for the risk of infectious disease

^b^Defined as ≥ 1 visit in the National Patient Register for infectious diseases listed in Table S1 in the Supplement

^c^Overall category includes ≥ 1 dispensation of an anti-mycobacterial or influenza antiviral medication in the Prescribed Drug Register in addition to visits. Upper only category includes ≥ 1 dispensation of influenza antiviral medications in addition to visits. Lower only category excludes respiratory tuberculosis

^d^Overall category includes ≥ 1 dispensation of an acne or herpes zoster medication in addition to visits. Acne only category includes ≥ 1 dispensation of an acne medication in addition to visits

**Fig. 2:**
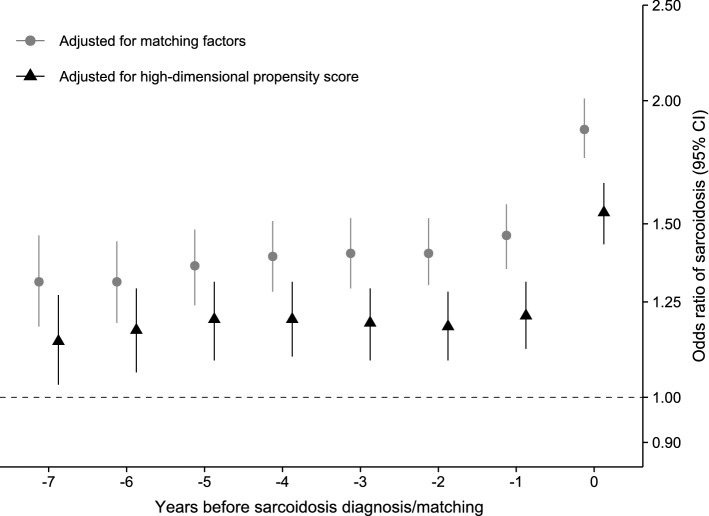
Odds ratios of sarcoidosis by latency period (temporal proximity of infectious disease to sarcoidosis diagnosis or matching; 0 years = no latency period, 1 year = ascertainment of infectious disease history started 1 year before sarcoidosis diagnosis or matching, etc.). Odds ratios were estimated using conditional logistic models adjusted for matching factors (birth year, sex, and residential location) and further controlled for deciles of a high-dimensional propensity score for the risk of infectious disease

Respiratory infections, which affected less than 7% of cases, were associated with 25% increased odds of sarcoidosis (aOR 1.25 [95% CI 1.10, 1.42]; Table 2). The aOR was lower (1.12) when only lower respiratory infections were considered (95% CI 0.93, 1.36). A 93% higher risk of sarcoidosis was observed in those with a history of ocular infection, but these infections were extremely rare (seen in < 1% of cases or controls). Skin, gastrointestinal, and genitourinary infections (except urinary tract infections that were used as negative control) conferred negligible risks for sarcoidosis development. Testing latency periods spanning from 1 to 7 years, we found that the aOR of sarcoidosis did not materially change except when no latency was used (Fig. 2 and Table S2; infection < 1 year before sarcoidosis diagnosis, aOR 1.54 [95% CI 1.43, 1.65]).

Cases treated for sarcoidosis were more likely to have a history of infection compared to cases who were not treated, resulting in a higher aOR of sarcoidosis in treated compared to not treated (1.41 [95% CI 1.25, 1.61] vs. 1.09 [95% CI 0.97, 1.23]; Table 3). Similarly, and despite low numbers, the OR associated with a history of infection was lower for Löfgren’s than for non-Löfgren’s disease, especially when no latency period was assumed (Table 3).

**Table 3:** Odds ratios of different sarcoidosis phenotypes (treated and non-treated disease at diagnosis, and Löfgren’s and non-Löfgren’s disease) associated with a history of infectious disease (≥ 1 visit in the National Patient Register)

	Individuals, n	History of infectious disease, n (%)	Odds ratio (95% CI)	
			Adjusted for matching factors	Adjusted for high-dimensional propensity score^a^
*No latency between infection history ascertainment and sarcoidosis diagnosis/matching*				
Löfgren’s syndrome^b^				
Cases	110	33 (30.0)	1.50 (0.96, 2.35)	1.52 (0.95, 2.43)
Controls	1100	250 (22.7)	1.00 [referent]	1.00 [referent]
Non-Löfgren’s disease^b^				
Cases	214	73 (34.1)	1.90 (1.40, 2.58)	1.77 (1.29, 2.43)
Controls	2135	463 (21.7)	1.00 [referent]	1.00 [referent]
*3*-*year latency between infection history ascertainment and sarcoidosis diagnosis/matching*				
Löfgren’s syndrome^b^				
Cases	110	21 (19.1)	1.06 (0.63, 1.78)	1.05 (0.61, 1.79)
Controls	1100	201 (18.3)	1.00 [referent]	1.00 [referent]
Non-Löfgren’s disease^b^				
Cases	214	40 (18.7)	1.14 (0.79, 1.65)	1.10 (0.75, 1.61)
Controls	2135	359 (16.8)	1.00 [referent]	1.00 [referent]
Treated sarcoidosis				
Cases	1713	407 (23.8)	1.68 (1.49, 1.89)	1.41 (1.25, 1.61)
Controls	17,108	2708 (15.8)	1.00 [referent]	1.00 [referent]
Non-treated sarcoidosis				
Cases	2362	439 (18.6)	1.21 (1.09, 1.36)	1.09 (0.97, 1.23)
Controls	23,580	3753 (15.9)	1.00 [referent]	1.00 [referent]

*CI* confidence interval

^a^Models for treated and non-treated sarcoidosis were adjusted for deciles of a high-dimensional propensity score for the risk of infectious disease. Due to the small sample size, models for Löfgren’s and non-Löfgren’s disease were adjusted for country of birth, education, salary, history of autoimmune disease, and number of relatives with history of autoimmune disease

^b^Information on Löfgren’s and non-Löfgren’s disease phenotype was available only for a subset of cases diagnosed by pulmonologists at Karolinska University Hospital in Stockholm

The aOR from the main analysis was markedly attenuated in sensitivity analyses for differential misclassification of the exposure (i.e. reverse causation; Table 4). Under the assumption that about one in 10 sarcoidosis cases might have been diagnosed with an infection because of preclinical disease, the OR for overall sarcoidosis was 1.02 (95% simulation interval 0.90, 1.15) and for treated sarcoidosis 1.20 (95% simulation interval 1.02, 1.44). Our findings were however robust to unmeasured confounding by smoking (OR 1.30 [95% simulation interval 1.18, 1.42]).

**Table 4:** Probabilistic bias analysis for differential misclassification of the exposure history of infectious disease (reverse causation bias)

Magnitude of misclassification	Median positive predictive value of infectious disease diagnosis (IQR), %		Odds ratio of sarcoidosis	
	Sarcoidosis cases	General population controls	Conventional estimate^a^ (95% CI)	Bias-adjusted estimate (95% SI)
*Analysis: sarcoidosis overall versus general population*				
Small	94.9 (93.5, 96.5)	100	1.19 (1.09, 1.29)	1.11 (0.98, 1.24)
Moderate	92.9 (91.5, 94.5)	100	1.19 (1.09, 1.29)	1.07 (0.95, 1.21)
Large	89.9 (88.5, 91.5)	100	1.19 (1.09, 1.29)	1.02 (0.90, 1.15)
*Analysis: treated sarcoidosis at diagnosis versus general population*				
Small	95.0 (93.4, 96.6)	100	1.41 (1.25, 1.61)	1.31 (1.12, 1.56)
Moderate	93.0 (91.4, 94.6)	100	1.41 (1.25, 1.61)	1.27 (1.08, 1.51)
Large	90.0 (88.4, 91.6)	100	1.41 (1.25, 1.61)	1.20 (1.02, 1.44)

*CI* confidence interval, *SI* simulation interval

^a^Adjusted for deciles of a high-dimensional propensity score for the risk of infectious disease

## Discussion

In this large register-based study, we observed a small increased odds ratio (OR) for developing sarcoidosis associated with a history of symptomatic infectious disease resulting in healthcare interaction. This weak association, which did not vary by time between infection and sarcoidosis diagnosis, attenuated completely when tested for reverse causation bias. Most of the observed risk was conferred by infections at sites related to sarcoidosis (e.g. respiratory), but associations with infections at unrelated sites (e.g. urinary tract infections) were also observed. We believe that these contradictory findings are a result of reverse causation bias induced by preclinical sarcoidosis, which may last for years in some cases before symptoms lead to sarcoidosis diagnosis.

At first glance, several of our findings (as summarized in Table 5) indicate that infections might play a role in the etiology of sarcoidosis. Specifically, we found a weak association between infectious disease and sarcoidosis, which persisted when several definitions were tested. Indeed, molecular studies reported ORs associated with infections, most prominently mycobacteria and propionibacteria, in the range of 9–20 [4, 6, 35, 36]. We could not replicate such high estimates even when we ascertained infectious disease history directly before sarcoidosis diagnosis aiming to mimic the timing of events in molecular studies. Direct comparisons with molecular studies are nevertheless challenging due to differences in exposure definitions and timing of exposure ascertainment.

**Table 5:** Points in favor and against the notion that infectious disease is a risk factor for sarcoidosis

*In favor*	
1.	An association exists between history of infectious disease and risk for sarcoidosis development in the future, although it is weak
2.	Infectious diseases at sites related to sarcoidosis (e.g. respiratory, ocular) are associated with sarcoidosis development in the future
*Against*	
1.	A history of infectious disease is rare among individuals with sarcoidosis, especially at sites related to the disease (e.g. respiratory and ocular infections). The population attributable fraction for infectious disease is estimated to be 3.3%
2.	Stronger associations for infectious diseases at difficult-to-diagnose sites of sarcoid inflammation (e.g. ocular disease) might suggest reverse causation
3.	Infections at sites not related to sarcoidosis or its complications (e.g. urinary tract infections) are associated with a risk of sarcoidosis development
4.	No dose–response relationship (or effect measure modification) by time since infectious disease diagnosis to sarcoidosis diagnosis
5.	Infectious disease associated with severe sarcoidosis (and non-Löfgren’s disease) might be a signal of reverse causation as those with severe disease are more likely to have a preclinical disease resulting in more infections due to a larger burden of immune disturbance
6.	In simulation analyses for reverse causation bias assuming one in ten diagnoses of infectious disease were influenced by preclinical sarcoidosis, the odds ratio of sarcoidosis was completely attenuated

We found a higher OR for treated sarcoidosis compared to non-treated sarcoidosis, and for non-Löfgren’s disease compared to Löfgren’s syndrome, a sarcoidosis phenotype associated with favorable prognosis. These observations indicate that infectious diseases might predispose to more severe sarcoidosis. Also, infections at two sites of prominent importance for the pathophysiology of sarcoidosis, the respiratory and ocular systems, were associated with sarcoidosis diagnosis in the future. This finding is in line with the hypothesis that an exogenous agent may enter the body through the respiratory tract or another tissue that is in direct contact with the environment [15].

Last, while this epidemiologic study is unique for sarcoidosis, studies using similar data reported rather stronger associations between infectious disease and several other autoimmune diseases, among others, Sjögren’s syndrome [22] and inflammatory myopathies [21].

Although at first glance findings in this study suggest that infectious disease is etiologically related to sarcoidosis development, we believe a more thorough scrutiny may lead to an alternative explanation—reverse causation bias. A likely mechanism for reverse causation could be an underlying immune disturbance due to preclinical (asymptomatic) sarcoidosis that spans years before diagnosis. In support of this notion, studies in sarcoidosis have shown that individuals appear to enter a diseased state characterized by sick leave absence, impaired productivity, visits to the doctor, and increased medication dispensing many years before diagnosis is established [26, 29, 30, 37]. This phenomenon, although not unique to sarcoidosis [38, 39], may be more pronounced here because of the phenotypic heterogeneity of the disease. Assuming reverse causation bias is present, is this bias large enough to account for all the observed association? As we have shown in bias analyses, even if less than one in 10 individuals who were diagnosed with sarcoidosis had preclinical disease, it would be enough to explain the association we observed in this study.

In addition to findings from bias analyses, many of the main results support this alternative explanation when more carefully interpreted (Table 5). First, the relative importance of (at least symptomatic) infection in causing sarcoidosis as indicated by the population attributable fraction is small (3.3%). Moreover, associations between infections at sites related to sarcoidosis were found to be of similar magnitude to unrelated sites (e.g. the urinary tract) suggesting there is no unique underlying pathophysiologic mechanism that could explain how one or more infectious agents cause sarcoidosis.

Second, and contrary to previous studies on the role of propionibacteria [6–9] or mycobacteria [4, 5], we did not identify any indications that these agents are implicated in the etiology of sarcoidosis. In the case of propionibacteria, we should acknowledge that acne is likely a misclassified proxy of infection by these commensal bacteria [40]. As for mycobacteria, we observed a higher proportion of patients with a history of respiratory tuberculosis in the sarcoidosis group (data not shown due to small numbers). A post hoc examination of their records in the patient register revealed that at the time of tuberculosis diagnosis they had either concomitant rheumatic disease or a difficult-to-diagnose organ involvement commonly associated with a delay in sarcoidosis diagnosis [37].

Third, ORs of sarcoidosis did not vary with time since infection (except when no latency period was used) suggesting that there was no time window during which an infectious agent might have triggered sarcoid inflammation. This finding is in line with our hypothesis that immune disturbance is present for years before sarcoidosis diagnosis. However, we should emphasize that this observation does not preclude the possibility that a pathogen was either dormant or acted more than 7 years before sarcoidosis was diagnosed, or even that infection was simply asymptomatic.

Last, assuming that individuals treated for sarcoidosis around diagnosis probably due to sarcoidosis severity are more likely to have a higher burden of immune disturbance compared to individuals with non-severe disease could explain the higher OR of treated sarcoidosis versus a very small increased OR of non-treated sarcoidosis. Based on the distinct genetics [41], seasonal variation [42], and acute onset of symptoms and favorable prognosis [28], one could hypothesize that Löfgren’s syndrome is more likely to be triggered by an infection. ORs for Löfgren’s syndrome, however, were lower than both sarcoidosis overall and non-Löfgren’s disease in particular, even when no latency period was used.

Our findings should be interpreted in light of the limitations of this study. In contrast to molecular studies, we could not explicitly identify specific pathogens; hence, the effect of one or more infectious agents may have been masked. To mitigate this issue, we used several definitions of infectious disease including healthcare visits and antimicrobial medication dispensations. Moreover, some misclassification of sarcoidosis is expected as we relied on ICD codes and results of histological examinations, bronchoscopies, or imaging were not available. As previously shown [3, 27], even large misclassification is unlikely to alter our inferences. Furthermore, because phenotyping sarcoidosis using ICD codes may lead to misclassification, we could not assess whether infections at a site were associated with sarcoid manifestations at the same site. Last, we cannot entirely preclude the possibility of unmeasured confounding being present (e.g. due to obesity). We believe, however, that because we used a rigorous algorithm to capture otherwise unmeasured confounders, residual confounding is likely small.

Despite the challenges, using prospectively collected population-based data provided us with the necessary power for robust inference, and most importantly, allowed us to address (at least in part) the issue of reverse causation bias and use methods to effectively capture otherwise unmeasured confounding. Finally, we believe that our findings are transportable to populations that are similar in terms of environmental exposures, genetics, and standard of care to the Swedish context.

## Conclusion

In this large case–control study we observed a weak association between symptomatic infectious disease and the development of sarcoidosis, which is identifiable years before sarcoidosis diagnosis. This association attenuated in bias analyses for reverse causation bias. Overall, we could not find enough evidence implicating common symptomatic infectious diseases in the etiology of the sarcoidosis. On the contrary, we believe that a latent immune disturbance might be the main reason behind the slightly higher burden of infectious disease observed before sarcoidosis diagnosis is established. Despite the challenges, large prospective studies with regular longitudinal examinations are warranted to further investigate this. Additionally, understanding the underlying immunologic mechanisms of this preclinical period is of critical significance to prevent or shorten the time to diagnosis and improve the prognosis of sarcoidosis. Until then, caution for reverse causation bias is required when using register data to define the exposure when the real disease onset is unknown.

## Electronic supplementary material

Below is the link to the electronic supplementary material.

Supplementary material 1 (DOCX 647 kb)
