# Supplementary material for: Are infectious diseases risk factors for sarcoidosis or a result of reverse causation? Findings from a population-based nested case–control study
Source: Eur J Epidemiol. 2020 Feb 11;35(11):1087–97. doi: 10.1007/s10654-020-00611-w (PMC7695666; doi:10.1007/s10654-020-00611-w)
Supplement: Supplementary file 1 — Supplementary material 1 (DOCX 647 kb) [file 10654_2020_611_MOESM1_ESM.docx]

| **SUPPLEMENT** |
| --- |

**Are infectious diseases risk factors for sarcoidosis or a result of reverse causation?
Findings from a population-based nested case-control study**

Marios Rossides, Susanna Kullberg, Johan Askling, Anders Eklund, Johan Grunewald,

Daniela Di Giuseppe, Elizabeth V. Arkema

Table of Contents

[SUPPLEMENTAL METHODS 2](#_Toc27469943)

[Statistical analysis 2](#_Toc27469944)

[Estimation of the population attributable fraction 2](#_Toc27469945)

[Estimation of the high-dimensional propensity scores 2](#_Toc27469946)

[Bias analysis 3](#_Toc27469947)

[Probabilistic bias analysis for differential exposure misclassification (reverse causation) 3](#_Toc27469948)

[Probabilistic bias analysis for unmeasured confounding by smoking 5](#_Toc27469949)

[References 6](#_Toc27469950)

[SUPPLEMENTAL TABLES 7](#_Toc27469951)

[Table S1. 7](#_Toc27469952)

[Table S2. 10](#_Toc27469953)

[SUPPLEMENTAL FIGURES 11](#_Toc27469954)

[Figure S1. 11](#_Toc27469955)

# SUPPLEMENTAL METHODS

## Statistical analysis

### Estimation of the population attributable fraction

We estimated the population attributable fraction [1] for the main exposure definition (≥1 visit for infectious disease) using the R package “AF” (version 0.1.5) [2]. Estimation was model-based and untransformed Wald 95% confidence intervals were reported. The estimand can be approximated by the formula $P_{Cinf}\left( 1-\frac{1}{aOR} \right)$, where $P_{Cinf}$ is the proportion of exposed cases and $aOR$ is the adjusted odds ratio of sarcoidosis. The estimation was based on the fair assumption that the outcome (sarcoidosis) is rare in the population (prevalence <10%) [3] so that the odds ratio closely approximates the risk ratio from a potential cohort study.

### Estimation of the high-dimensional propensity scores

We estimated a high-dimensional propensity score for each analysis separately using a semi-automated algorithm described by Schneeweiss and colleagues [4]. The following dimensions of register-based data were considered for the construction of the propensity score:

- Inpatient visit data classified using International Classification of Disease codes (ICD; Swedish 10^th^ revision) from the National Patient Register available since 1999. Granularity was set to the three-digit ICD code.
- ICD-10-coded outpatient visits from the National Patient Register available since 2001 (three-digit granularity).
- Visits for day surgery and other procedures coded using Swedish operational codes (Klassifikation av vårdåtgärder, KVÅ) in the National Patient Register available since 1999 (five-digit granularity).
- Dispensation of prescribed medications identified through Anatomical Therapeutic Chemical (ATC) Classification System codes in the Prescribed Drug Register available since Jul 1, 2005 (seven-digit granularity).

To select covariates from the four data dimensions, we followed the procedure outlined below:

1. For analyses assuming a latency period of three years, visits, operations, and dispensations were restricted to the interval four to six years before index date. Two years were enough time to capture chronic morbidity in all patients irrespective of the year of sarcoidosis diagnosis or matching (given data availability).
2. ICD codes for infectious diseases and ATC codes for antimicrobials were excluded.
3. The 200 most prevalent codes were identified from each component (n=200).
4. Three covariates for each of these codes were created indicating whether each code appeared once during the aforementioned two-year interval, or more than the median, or more than the 75^th^ percentile.
5. Covariates were ranked in descending order according to the strength of the association between each covariate and the odds of infectious disease and the top 500 were retained for score estimation models (k=500).

In addition to the 500 covariates selected using the procedure above, indicators for the utilization of healthcare and several predefined confounding variables were also added to the logistic regression models that were used to estimate the propensity score for history of infectious disease: age at sarcoidosis diagnosis/matching, sex, region of residence, birth country, education, annual gross salary, civil status, history of autoimmune disease or immunodeficiency (excluding acquired immunodeficiency), number of first degree relatives with history of autoimmune disease, and year of sarcoidosis diagnosis/matching.

The logistic regression model was fitted to the sample of controls to obtain unbiased coefficients accounting for the biased sampling frame of our study (case-heavy sampling) [5]. Coefficients obtained from the controls-based model were then used to estimate the probability of infectious disease for both cases and controls, which was then categorized into deciles. For the propensity score-adjusted models, 95% confidence intervals were constructed using sandwich-variance estimators, which proved to have comparable error coverage to bootstrapping techniques but much faster computation time.

## Bias analysis

### Probabilistic bias analysis for differential exposure misclassification (reverse causation)

We used probabilistic bias analysis methods [6,7] to check the robustness of our primary estimate for the association between infectious disease and sarcoidosis development in the presence of differential misclassification of the exposure (≥1 visit for infection). We assumed that preclinical sarcoidosis, which may be present for years before diagnosis is eventually determined might increase the risk of developing an infection (and subsequently being diagnosed with an infectious disease) in these individuals. This is a case for reverse causation bias or independent and differential exposure misclassification. The directed acyclic graph below depicts how this independent and differential misclassification of exposure ascertainment may affect the association between infectious disease and sarcoidosis. For completeness, the graph also shows non-differential independent misclassification of the outcome (sarcoidosis identified using register data; in grey color). For simplicity, we assumed that it is not present as it does markedly influence our analysis in terms of reverse causation bias.


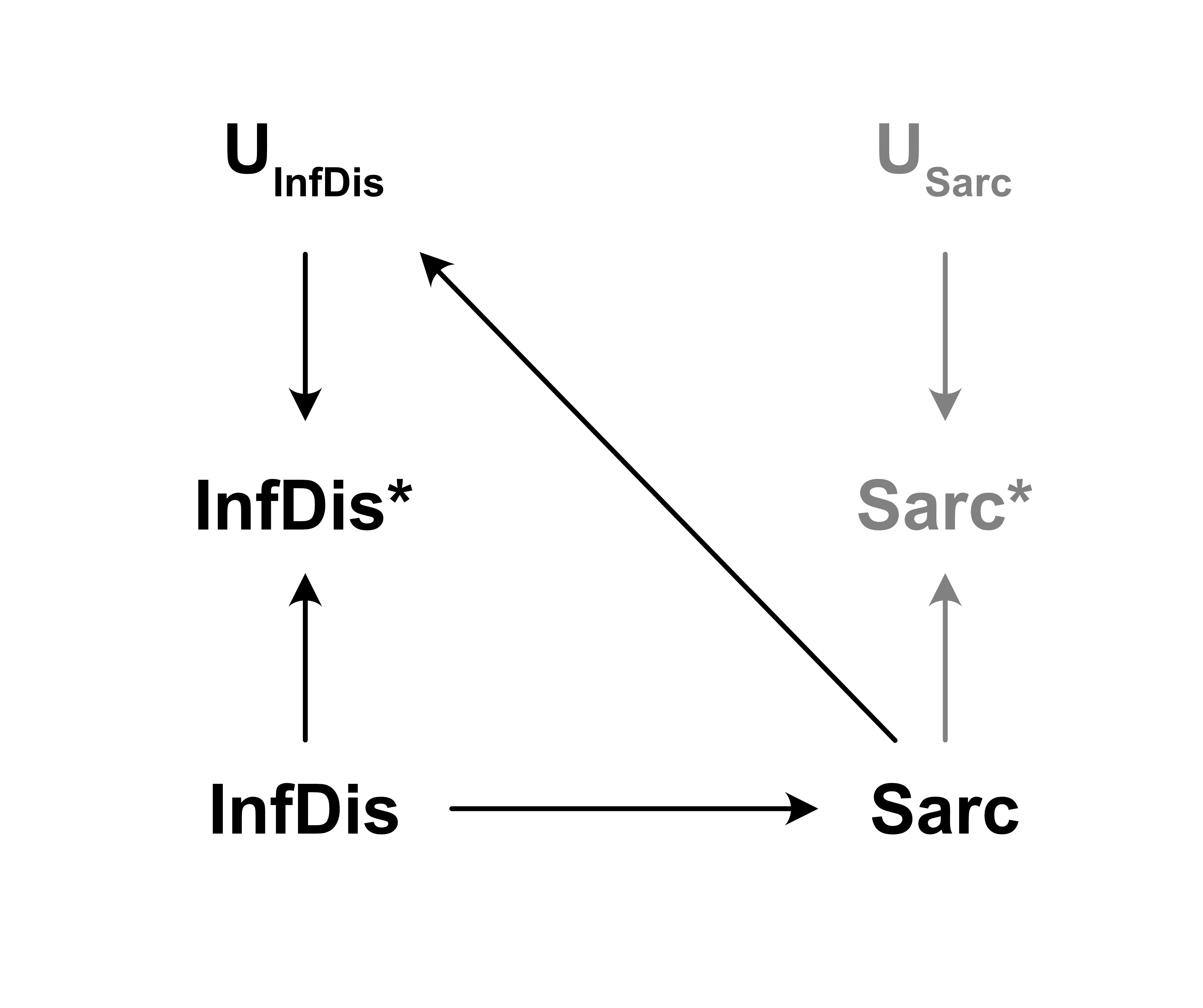


In this study, we were interested in quantifying the causal pathway from the exposure (**InfDis** in the figure above) to the outcome (**Sarc**). We only had data on a misclassified version of the exposure (**InfDis***), which we believe was influenced by an unknown process (denoted as **U_InfDis_**), which, in turn, was influenced by preclinical sarcoidosis (e.g. an undiagnosed immune disturbance due to sarcoidosis). The estimated measure of association in this study reflected a combination of the following (open) paths: (a) **InfDis*** ← **InfDis** → **Sarc** and (b) **InfDis*** ← **U_InfDis_** ← **Sarc**. The first path (a) was of interest, whereas the second (b) was a biasing path (i.e. the reverse causation/misclassification path).

To test the impact of reverse causation bias (or independent differential misclassification of the exposure), we developed three simulation scenarios assuming a small, moderate, and large magnitude of misclassification. Because it was not feasible to acquire data that could directly inform us on the actual magnitude of exposure misclassification in cases, we chose to estimate the association between infectious disease and sarcoidosis under three bias scenarios of varying magnitude of misclassification. We considered a magnitude of 10% misclassification (i.e. one in 10 cases could be diagnosed with infectious disease due to preclinical sarcoidosis) to reflect large exposure misclassification. We based this estimate our previous observation that approximately one in 10 individuals with sarcoidosis had a history of healthcare visits related to preclinical sarcoidosis (e.g. uveitis) several years before diagnosis [8]. The magnitude of misclassification was determined by the positive and negative predictive values of the exposure definition in sarcoidosis cases and general population controls separately.

Values for the positive and negative predictive values were sampled from the distributions defined as follows:

| **Magnitude of differential misclassification** | **Sarcoidosis cases** | |  | **General population controls** | |
| --- | --- | --- | --- | --- | --- |
|  | **Positive predictive value*** | **Negative predictive value** |  | **Positive predictive value** | **Negative predictive value** |
| Small | Triangular  (0.90, 1.00, 0.95) | 1 |  | 1 | 1 |
| Moderate | Triangular  (0.88, 0.98, 0.93) | 1 |  | 1 | 1 |
| Large | Triangular  (0.85, 0.95, 0.90) | 1 |  | 1 | 1 |
| *The triangular distribution parameters are defined as: Triangular(*a*=min, *b*=max, *c*=mode) | | | | | |

We performed 1000 Monte Carlo simulations for each of the three bias scenarios to obtain an odds ratio of sarcoidosis corresponding to the one from the main analysis, but ‘corrected’ for measurement error. We then bootstrapped our analyses to obtain a (percentile) 95% simulation interval accounting for the systematic and random errors from the main analysis.

### Probabilistic bias analysis for unmeasured confounding by smoking

We performed a similar simulation to check whether the odds ratio from the main analysis was robust in the presence of confounding by current smoking for which data we did not have. We defined the bias parameters as follows:

- Prevalence of current smoking in the exposed: Uniform (min=0.35, max=0.55).
- Prevalence of current smoking in the unexposed: Uniform (min=0.15, max=0.35).
- Association between current smoking and sarcoidosis: Uniform (min=0.03, max=0.06), which translates into an odds ratio of approximately 0.70).

The definitions of the bias parameters were informed by prior research [9–11] and data on smoking habits in the general population obtained from national health surveys [12]. To estimate a corrected-for-smoking odds ratio of sarcoidosis, we repeatedly sampled from the above distributions and calculated the total probability of the unmeasured confounder for each individual based on their exposure and outcome status. The binary unknown confounder was determined for each individual by Bernoulli trials based on their aforementioned probability. We re-estimated the odds ratio from the main analysis using conditional logistic regression and obtained a 95% (percentile) non-parametric simulation interval accounting for the systematic and random errors from the main analysis using bootstrap techniques.

## References

1. Bruzzi P, Green SB, Byar DP, Brinton LA, Schairer C. Estimating the population attributable risk for multiple risk factors using case-control data. Am J Epidemiol. 1985;122:904–14.

2. Dahlqwist E, Sjölander A. AF: Model-based estimation of confounder-adjusted attributable fractions. R package version 0.1.5. (https://CRAN.R-project.org/package=AF). Stockholm; 2019.

3. Arkema E V, Grunewald J, Kullberg S, Eklund A, Askling J. Sarcoidosis incidence and prevalence: a nationwide register-based assessment in Sweden. Eur Respir J. 2016;48:1690–9.

4. Schneeweiss S, Rassen JA, Glynn RJ, Avorn J, Mogun H, Brookhart MA. High-dimensional propensity score adjustment in studies of treatment effects using health care claims data. Epidemiology. 2009;20:512–22.

5. Månsson R, Joffe MM, Sun W, Hennessy S. On the estimation and use of propensity scores in case-control and case-cohort studies. Am J Epidemiol. 2007;166:332–9.

6. Lash TL, Fox MP, Fink AK. Applying Quantitative Bias Analysis to Epidemiologic Data. New York City, NY: Springer; 2009.

7. Fox MP, Lash TL, Greenland S. A method to automate probabilistic sensitivity analyses of misclassified binary variables. Int J Epidemiol. 2005;34:1370–6.

8. Rossides M, Kullberg S, Eklund A, Grunewald J, Arkema E V. Sarcoidosis diagnosis and treatment in Sweden: A register-based assessment of variations by region and calendar period. Respir Med. 2020;161:105846.

9. Gupta D, Singh AD, Agarwal R, Aggarwal AN, Joshi K, Jindal SK. Is tobacco smoking protective for sarcoidosis? A case-control study from North India. Sarcoidosis Vasc Diffus Lung Dis. 2010;27:19–26.

10. Ungprasert P, Crowson CS, Matteson EL. Smoking, obesity and risk of sarcoidosis: A population-based nested case-control study. Respir Med. 2016;120:87–90.

11. Arcavi L, Benowitz NL. Cigarette smoking and infection. Arch Intern Med. 2004;164:2206.

12. Folkhälsomyndigheten. Nationella folkhälsoenkäten-tobaksvanor [National public health survey-smoking habits]. 2016 [cited 2018 Jun 14]. Available from: https://www.folkhalsomyndigheten.se/folkhalsorapportering-statistik/om-vara-datainsamlingar/nationella-folkhalsoenkaten/

# SUPPLEMENTAL TABLES

Table S1. International Classification of Disease (ICD) and Anatomical Therapeutic Chemical (ATC) Classification System codes used to identify visit for in the National Patient Register and medication dispensations in the Prescribed Drug Register.

| **Disease** | **Swedish International Classification of Disease (ICD) codes*** | | | **Anatomical Therapeutic Chemical (ATC) Classification System codes** |
| --- | --- | --- | --- | --- |
|  | **10^th^ revision** | **9^th^ revision** | **8^th^ revision** |  |
| Respiratory infections (overall) | A06.5; A15; A16; A24; A31.0; A36.0; A36.1; A36.2; A37; A38; A42.0; A43.0; A54.5; A56.4; B38; B39; B40–B49; A70; J00–J18; J20–J22; J34.0; J39.0; J39.1; J85; J86 |  |  | J04A; J05AH |
| Upper respiratory infections | A24; A36.0; A36.1; A36.2; A37; A38; A54.5; A56.4; J00–J11; J34.0; J39.0; J39.1 |  |  | J05AH |
| Lower respiratory infections | A06.5; A31.0; A42.0; A43.0; A70; B38; B39; B40–B49; J12–J18; J20–J22; J85; J86 |  |  |  |
| Respiratory tuberculosis | A15; A16 |  |  | J04A |
| Skin infections (overall) | A06.7; A18.4; A26.0; A30; A31.1; A32.0; A36.3; A43.1; A44.1; A46; A65; A66; A67; B00–B09; B35; B36; B85–B87; H60.0; H60.1; L00–L08; L70.0; N73.0; N73.1; N73.2 |  |  | D10; J05AB01; J05AB11 |
| Acne | L70.0 |  |  | D10 |
| Ocular infections | A18.5; A54.3; A71; B30; H10.0; H22.0; H32.0 |  |  |  |
| Gastrointestinal infections | A00; A01; A02.0; A03; A04; A05.0; A06.0; A06.1; A06.2; A06.3; A06.4; A07–A09; A18.3; A42.1; A54.6; A56.3; B15–B19; B70–B79; B80; B81; B82; B88; J36; K11.3; K12.2; K61; K63.0; K75.0; K77.0; K93.0; K93.1 |  |  |  |
| Genitourinary infections (overall) | A18.1; A51; A54.0; A54.1; A54.2; A55; A56.0; A56.1; A56.2; A57–A59; A60.0; A60.1; A60.9; A63.0; A63.8; N10; N11; N22.0; N29.0; N30.0; N34.0; N37.0; N39.0; N41; N45; N48.1; N70–N72; N73.3; N73.4; N75.1; N75.8; N76; N77.0; N77.1 |  |  |  |
| Urinary tract infections | N30.0; N39.0 |  |  |  |

**Table S1.** (Continued).

| **Disease** | **Swedish International Classification of Disease (ICD) codes*** | | | **Anatomical Therapeutic Chemical (ATC) Classification System codes** |
| --- | --- | --- | --- | --- |
|  | **10^th^ revision** | **9^th^ revision** | **8^th^ revision** |  |
| Other infections | A02.1; A02.2; A02.8; A02.9; A05.1–A05.4; A05.8; A05.9; A06.6; A06.8; A06.9; A17; A18.0; A18.2; A18.6; A18.7; A18.8; A19; A20–A23; A25; A26.7; A26.8; A26.9; A27; A28; A31.8; A31.9; A32.1; A32.7; A32.8; A32.9; A33–A35; A36.8; A36.9; A39.0; A39.1–A39.5; A39.8; A39.9; A40; A41; A42.2; A42.7; A42.8; A42.9; A43.8; A43.9; A44.0; A44.8; A44.9; A48; A49; A50; A52; A53; A54.4; A54.8; A54.9; A56.8; A64; A68; A74; A75; A77–A99; B20–B27; B33; B34; B37; B50–B59; B60–B69; B83; B89; B90; B91; B92; B94.0; B94.1; B94.2; B94.8; B94.9; B95; B96; B97; B99; G00–G02; G04.2; G05–G07; H67.0; H67.1; H70.0; H70.1; H75.0; I30.1; I32.0; I32.1; I33.0; I40.0; I41.0; I41.1; I41.2; I68.1; I98.0; I98.1; M00; M01; M60.0; M63.0; M63.1; M63.2; N61; N73.5; N73.9; R57.2; R65; R65.0; R65.1 |  |  |  |
| Sarcoidosis | D86 | 135 | 135 |  |
| In-need-of-treatment sarcoidosis |  |  |  | H02AB01; H02AB02; H02AB04; H02AB06; H02AB07; L01BA01; L04AX01; L04AX03 |
| Hematopoietic and lung malignancy |  |  | **7^th^ ICD revision:**  162, 163, 200‒205 |  |

**Table S1.** (Continued).

| **Disease** | **Swedish International Classification of Disease (ICD) codes*** | | | **Anatomical Therapeutic Chemical (ATC) Classification System codes** |
| --- | --- | --- | --- | --- |
|  | **10^th^ revision** | **9^th^ revision** | **8^th^ revision** |  |
| Autoimmune disease | D59.1; D69.3; E06.3; E05.0; E10; E27.1; J45; J46; K50; K51; K90.0; K74.3; L63; L10; L12; L40; M0; M31.3; M31.6; M31.5; M32.1; M32.8; M32.9; M33; M34; M35.0; M35.1; M35.2; M45; G70.0; G35; G61.0; D51.0; D68.6; D86*  *Sarcoidosis ICD-codes were used for identifying disease in first-degree relatives only | 283A; 287D; 245C; 242A; 255E; 555; 556; 579A; 704A; 694E; 694F; 696; 714; 446E; 446F; 493; 710; 136B; 720; 358A; 340; 357A; 281A; 135* | 283,90; 287,10; 245,30; 242,00; 255,10; 563; 269,10; 704,00; 694; 696; 446,20; 446,30; 446,38; 493; 734,1; 716; 734,0; 734,9; 136,07; 712,4; 733,00; 340; 357; 281,0; 135* |  |
| Immunodeficiency (excluding acquired) | D83; D80; D81; D82; D84 | 279J; 279L; 279M; 279X |  |  |
| *The Swedish ICD classification system’s 10^th^ revision was in use starting 1997, the 9^th^ revision between 1987 and 1996 and the 8^th^ revision between 1969 and 1986. | | | | |

Table S2. Relative risk of sarcoidosis associated with a history of infectious disease stratified by recency of infectious disease to sarcoidosis diagnosis or matching.

|  | **History of infectious disease, n (%)** | |  | **Relative risk of sarcoidosis**  **(95% CI)** | |
| --- | --- | --- | --- | --- | --- |
|  | **Sarcoidosis cases**  (n=4075) | **General population controls**  (n=40 688) |  | **Adjusted for matching factors** | **Adjusted for high-dimensional propensity score*** |
| **Time from infection to sarcoidosis/matching** |  |  |  |  |  |
| 0 years | 1364 (33.5) | 8764 (21.5) |  | 1.87 (1.75, 2.01) | 1.54 (1.43, 1.65) |
| ≥1 year | 1062 (26.1) | 8009 (19.7) |  | 1.46 (1.35, 1.57) | 1.21 (1.12, 1.31) |
| ≥2 years | 939 (23.0) | 7216 (17.7) |  | 1.40 (1.30, 1.52) | 1.18 (1.09, 1.28) |
| ≥3 years (main analysis) | 846 (20.8) | 6461 (15.9) |  | 1.40 (1.29, 1.52) | 1.19 (1.09, 1.29) |
| ≥4 years | 752 (18.5) | 5743 (14.1) |  | 1.39 (1.28, 1.51) | 1.20 (1.10, 1.31) |
| ≥5 years | 650 (16.0) | 5030 (12.4) |  | 1.36 (1.24, 1.48) | 1.20 (1.09, 1.31) |
| ≥6 years | 538 (13.2) | 4266 (10.5) |  | 1.31 (1.19, 1.44) | 1.17 (1.06, 1.29) |
| ≥7 years | 447 (11.0) | 3509 (8.6) |  | 1.31 (1.18, 1.46) | 1.14 (1.03, 1.27) |
| CI = confidence interval.  *Adjusted for deciles of a high-dimensional propensity score for the risk of infection. | | | | | |

# SUPPLEMENTAL FIGURES


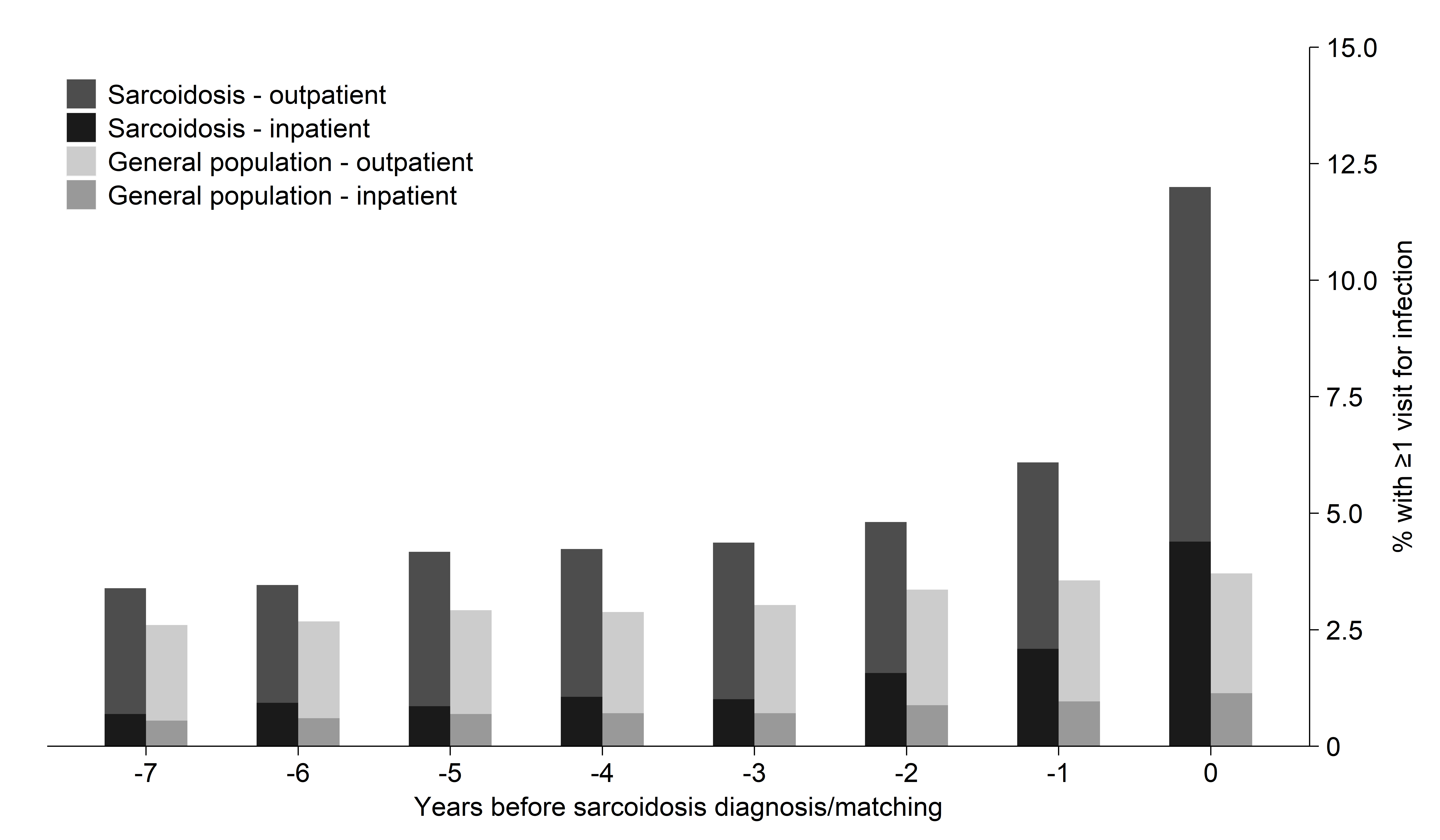


Figure S1. Proportion of cases and controls with at least one visit for infectious disease by time to sarcoidosis diagnosis or matching in years. Inpatient and outpatient visits in the National Patient Register were plotted separately.
